# Artificial Intelligence-Powered Left Ventricular Ejection Fraction Analysis Using the LVivoEF Tool for COVID-19 Patients

**DOI:** 10.3390/jcm12247571

**Published:** 2023-12-08

**Authors:** Ziv Dadon, Yoed Steinmetz, Nir Levi, Amir Orlev, Daniel Belman, Adi Butnaru, Shemy Carasso, Michael Glikson, Evan Avraham Alpert, Shmuel Gottlieb

**Affiliations:** 1 Jesselson Integrated Heart Center, Shaare Zedek Medical Center, Jerusalem 9103102, Israel; 2 Faculty of Medicine, Hebrew University of Jerusalem, Jerusalem 9112102, Israel; 3 Intensive Care Unit, Shaare Zedek Medical Center, Jerusalem 9103102, Israel; 4 The Azrieli Faculty of Medicine, Bar-Ilan University, Zefat 1311502, Israel; 5 Department of Emergency Medicine, Shaare Zedek Medical Center, Jerusalem 9103102, Israel; 6 Sackler Faculty of Medicine, Tel Aviv University, Tel Aviv 6997801, Israel

**Keywords:** artificial intelligence (AI), COVID-19, hand-held echocardiogram, prognosis, ventricular function, left

## Abstract

We sought to prospectively investigate the accuracy of an artificial intelligence (AI)-based tool for left ventricular ejection fraction (LVEF) assessment using a hand-held ultrasound device (HUD) in COVID-19 patients and to examine whether reduced LVEF predicts the composite endpoint of in-hospital death, advanced ventilatory support, shock, myocardial injury, and acute decompensated heart failure. COVID-19 patients were evaluated with a real-time LVEF assessment using an HUD equipped with an AI-based tool vs. assessment by a blinded fellowship-trained echocardiographer. Among 42 patients, those with LVEF < 50% were older with more comorbidities and unfavorable exam characteristics. An excellent correlation was demonstrated between the AI and the echocardiographer LVEF assessment (0.774, *p* < 0.001). Substantial agreement was demonstrated between the two assessments (kappa = 0.797, *p* < 0.001). The sensitivity, specificity, PPV, and NPV of the HUD for this threshold were 72.7% 100%, 100%, and 91.2%, respectively. AI-based LVEF < 50% was associated with worse composite endpoints; unadjusted OR = 11.11 (95% CI 2.25–54.94), *p* = 0.003; adjusted OR = 6.40 (95% CI 1.07–38.09, *p* = 0.041). An AI-based algorithm incorporated into an HUD can be utilized reliably as a decision support tool for automatic real-time LVEF assessment among COVID-19 patients and may identify patients at risk for unfavorable outcomes. Future larger cohorts should verify the association with outcomes.

## 1. Introduction

Given the well-established association between the cardiovascular system and COVID-19 disease, an echocardiogram may have an important role in patient evaluation [1,2,3]. Indeed, recently conducted large observational studies have reported the high prevalence of left and right ventricular dysfunction among almost one-third of a cohort of critically ill COVID-19 patients with different phenotypes of RV involvement [4,5]. Nonetheless, routine echocardiography for all COVID-19 patients is currently discouraged due to concerns of infection control, equipment contamination, and excessive workload in the setting of a pandemic [6,7]. A pragmatic approach to the increased demand for ultrasound usage for cardiovascular and pulmonary assessment is a hand-held ultrasound device (HUD), which is highly feasible with good quality and can be easily cleaned and dedicated to a specific ward [8,9]. The use of HUDs in COVID-19 cases was also found to lead to a significant reduction in the scanning time and the total duration of time spent in the patient’s room alongside good battery usage and reasonable operator-to-patient proximity [8,10,11]. An abnormal echocardiogram, including reduced left ventricular ejection fraction (LVEF) and moderate/severe valvular abnormalities, using HUDs in COVID-19 patients was associated with a higher proportion of comorbidities and independently predicted major adverse outcomes [12]. This may therefore be used as an important risk stratification tool among high-risk COVID-19 patients (oxygen saturation < 94%) [12]. However, HUDs have several limitations, including small screen sizes, limited image quality, a lack of advanced measurements, intermediate battery lives, and unclear findings which need to be confirmed by an official high-end device [13]. These challenges, as well as strict COVID-19 precautions, may affect the real-time diagnosis accuracy of left ventricular (LV) systolic function. Artificial intelligence (AI) is now increasingly used for imaging purposes, specifically including LV function assessment in the hands of experienced operators as well as novice users for clinical purposes [14]. As such, it may also be utilized as a decision support tool in the COVID-19 setting for ventricular assessment and for the potential screening of patients.

The objective of this study is to assess the diagnostic accuracy of an AI-based assessment tool using HUDs as compared with expert echocardiographers in evaluating the LVEF among COVID-19 patients and to examine whether AI-based reduced LVEF (<50%) is associated with patient outcomes.

## 2. Materials and Methods

### 2.1. Study Setting

This is a prospective study of real-time focused echocardiogram using an HUD conducted on PCR-confirmed non-selected COVID-19 patients hospitalized in designated wards at a tertiary medical center (Shaare Zedek Medical Center, Jerusalem, Israel) from 28 April through 26 July 2020. The study was approved by the hospital’s Institutional Review Board (IRB; 0138-20-SZMC, approved on 19 April 2020). 

### 2.2. Study Endpoints

The primary endpoint of the study included the LVEF assessment accuracy by the AI-based tool as compared to a fellowship-trained echocardiographer of HUD clips among patients hospitalized with COVID-19. The secondary endpoint outcome measures included the composite of in-hospital death, advanced ventilatory support (high-flow nasal cannula, non-invasive positive airway pressure support, and invasive ventilation), shock, myocardial injury (defined as >3 times the upper normal limit of high-sensitivity cardiac troponin-I (hs-cTnI)), and acute decompensated heart failure (ADHF). Other secondary endpoint measures included venous thromboembolism, anti-COVID-19 drug use, sepsis, and length of hospital stay. The anti-COVID-19 drugs included dexamethasone (when used for this indication), azithromycin, hydroxychloroquine, remdesivir, opaganib, and COVID-19 convalescent plasma.

### 2.3. Study Protocol

**Figure 1:**
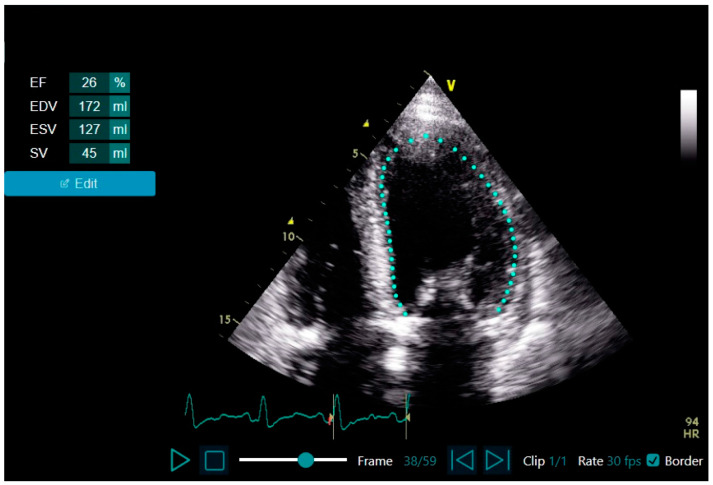
The LVivo EF: AI-based tool for automated LVEF assessment from apical 4-chamber view echocardiographic clips. Abbreviations. AI, artificial intelligence; LVEF, left ventricular ejection fraction.

All echocardiographic clips were acquired by cardiologists or intensivists with no prior dedicated fellowship training in echocardiography and with at least one year of routine echocardiogram acquisition as part of their practice. They wore personal protective equipment including a full gown, N-95 face mask, face screen, and two-to-three sets of gloves. Per the IRB approval, conscious patients provided informed consent verbally, and those not able to consent underwent an echocardiogram if clinically indicated. The echocardiogram was performed within 48 h of their hospitalization using an HUD (Vscan Extend with Dual Probe; General Electric, Boston, MA, USA) equipped with LVivo EF (DiA Imaging Analysis Ltd., Be’er Sheva, Israel; Figure 1), a program which uses AI to provide the automated calculation of LVEF from the apical 4-chamber (A4ch) view. This tool provides different LV parameters, including LVEF, LV end diastolic and systolic volume, and stroke volume.

Upon exam completion, technical variables of the study were recorded, including heart rate (in beats per minute), the mean distance between the operator and the head of the patient (in centimeters; from the chin of the operator to the nose of the patient), length of study (in minutes), battery usage (in percentage change), and successful completion of all echocardiogram views. As was previously mentioned, COVID-19 can result in ventricular dysfunction, and as such, we included all patients with and without prior comorbidities in the study, including those with previously known heart failure with preserved ejection fraction.

### 2.4. Echocardiogram Acquisition and LVEF Assessment Using LVivo EF vs. Expert Assessment

The operators performed the focused echocardiography examination using the HUD and acquired the echocardiography clips from the following views: parasternal long-axis, parasternal short-axis, A4Ch, apical 2-chamber, and substernal views (including longitudinal and IVC views). If possible, an apical 3-chamber view was added as well. For the A4Ch views, the operators were instructed to focus on the LV, optimizing the clip and refraining from foreshortening. The interventricular septum was aligned parallel to the plane and a heart cycle of at least 2 beats was acquired. The depth was modified to ensure that the LV encompassed two-thirds of the view. The acquired clips were then assessed on-line for LVEF using the AI-based tool and sent wirelessly to a picture archiving platform (McKesson Cardiology™, version 14.0, San Diego, CA, USA). In the case of a failure of the AI tool to automatically calculate the LVEF or if the border tracings were incorrect, the image acquisition was repeated (up to four subsequent failures). Next, the acquired clips were assessed by a certified echocardiographic technician (equivalent to a Registered Diagnostic Cardiac Sonographer in the United States) for a second LVEF measurement using Simpson’s method, as well as being visually evaluated by an experienced cardiologist (A.B.), blinded to the AI-based tool’s results. The echocardiographer also assessed the clips for image quality according to a 3-categories scale (good—optimal visualization; fair—proper visualization of >50% of the segments; and poor—inappropriate visualization). Those who refused to participate as well as those with an unsuccessful AI automatic measurement were excluded from the study (as the accuracy of the AI-based tool and its association with outcomes cannot be addressed regarding these studies). Variables including demographics and past medical history were obtained from the electronic charts.

### 2.5. Sample Size Calculation

Sample size calculations were designed to meet the study primary endpoint and were performed using G*Power software (version 3.1.9.4, Heinrich Heine University Düsseldorf, Düsseldorf, Germany). We planned a paired study with LVEF comparison and a 1:1 ratio between the AI-based tool and the expert echocardiographer assessments. Based on previous data regarding LVivo EF accuracy [15], we assumed an effect size of 0.5 between the AI-based tool LVEF calculation and the echocardiographer assessment. Based on these assumptions, we calculated that data analyzed from 37 participants would suffice to reject the null hypothesis with a probability (power) of 0.9. The type I error was calculated as 0.05 and was two-sided.

### 2.6. Statistical Analyses

Descriptive statistics were used to analyze baseline and clinical characteristics as well as echocardiogram results and endpoints. Chi-square or Fisher’s exact tests were used for categorical variables, and the t-test or Mann–Whitney U test was used for continuous variables. Test selection was based on data distribution and normalcy. Study participants were divided into preserved or reduced LVEF (LVEF < 50%) based on their AI tool results. Continuous LVEF assessments were compared for linear correlation using Pearson’s correlation coefficient. For categorical variables, the inter-rater reliability using the Kappa coefficient was then calculated. The LVEF measurements were transformed into dichotomous variables to enable Kappa calculation using LVEF cutoffs of 50% (i.e., normal/preserved vs. reduced LVEF). The ability of the AI-based tool to identify patients with LVEF < 50% was then tested for sensitivity, specificity, and positive and negative predictive values (PPV and NPV, respectively).

The unadjusted odds ratio (OR) and 95% confidence interval (CI) were calculated to test the associations between reduced LVEF and the composite and individual endpoints. The odds ratio was defined as the ratio of the odds of the endpoint in the presence of reduced LVEF and the odds of the endpoint in the absence of reduced LVEF (i.e., the presence or absence of LVEF < 50% and the endpoint).

Multivariate logistic regression analysis (OR and 95% CI) was calculated for the association between reduced LVEF and the composite endpoint, including age and combined pertinent comorbidities variable (baseline characteristics covariates with *p* values of less than 0.05). Statistical analysis was performed using SP Statistics for Windows version 21 (SPSS Inc., Chicago, IL, USA). All tests were two-sided, with a *p*-value ≤ 0.05 considered statistically significant.

## 3. Results

A total of 56 patients were initially included in the study. Four patients refused to participate and thus were excluded. The AI automatic measurement was not successful in 10 patients, leaving 42 (75.0%) patients for inclusion in the analysis. 

### 3.1. Baseline, Clinical, and Test Characteristics and in-Hospital Course: AI-Based Preserved vs. Reduced LVEF (Table 1)

Among 42 patients, 21 (50%) were males with a mean age of 53.3 ± 17.8 years and a mean BMI of 27.6 ± 5.1 kg/m^2^. Seven (16.7%) patients could not turn on their left side, and three (7.1%) were not proficient in maintaining effective communication (i.e., adhering to instructions and cooperating with the examination). The mean duration of the echocardiogram studies was 6.8 ± 2.2 min, battery usage was 13.1 ± 4.9%, and the mean operator-to-patient distance was 64.5 ± 9.3 cm.

As compared with patients with preserved LVEF, those with reduced LVEF (<50%) were significantly older (63.5 ± 16.3 vs. 49.7 ± 17.1 years), had a higher proportion of comorbidities (including a history of hypertension, hyperlipidemia, ischemic heart disease, past revascularization, and heart failure) and were more frequently treated with heart failure guideline-directed medical therapy, anti-platelets, and statins. Patients with reduced LVEF were more likely to present with shortness of breath; had a higher proportion of atrial fibrillation/flutter rhythm; and had higher levels of creatinine, hs-cTnI, C-reactive protein, and D-dimer but lower nadir levels of albumin. Concerning exam characteristics and technical aspects, patients with reduced LVEF had a longer exam as well as closer proximity to the examiner.

**Table 1:** Baseline and clinical characteristics: preserved vs. reduced LVEF as per the AI-based tool.

Variable	All *n* = 42	Preserved LVEF *n* = 31	Reduced LVEF *n* = 11	*p*-Value
Baseline characteristics				
Age, mean ± SD	53.3 ± 17.8	49.7 ± 17.1	63.5 ± 16.3	0.026
Male, *n* (%)	21 (50.0)	15 (48.4)	6 (54.5)	0.726
Body mass index, mean ± SD	27.6 ± 5.1	27.1 ± 4.3	28.7 ± 6.9	0.459
Smoking, *n* (%)	4 (9.5)	3 (9.7)	1 (9.1)	1.000
Diabetes mellitus, *n* (%)	14 (33.3)	8 (25.8)	6 (54.5)	0.082
Hypertension, *n* (%)	13 (31.0)	5 (16.1)	8 (72.7)	0.001
Hyperlipidemia, *n*(%)	13 (31.0)	5 (16.1)	8 (72.7)	0.001
Ischemic heart disease, *n* (%)	7 (16.7)	2 (6.5)	5 (45.5)	0.009
Cerebrovascular accident, *n* (%)	1 (2.4)	1 (3.2)	0	1.000
Revascularization, *n* (%)	7 (16.7)	2 (6.5)	5 (45.5)	0.009
Heart failure, *n* (%)	5 (11.9)	1 (3.2)	4 (36.4)	0.013
Valve replacement, *n* (%)	2 (4.8)	1 (3.2)	1 (9.1)	0.460
CIED, *n* (%)	1 (2.4)	0	1 (9.1)	0.262
Cognitive decline, *n* (%)	4 (9.5)	2 (6.5)	2 (18.2)	0.277
Debilitation, *n* (%)	6 (14.3)	3 (9.7)	3 (27.3)	0.314
Chronic lung disease, *n* (%)	3 (7.1)	3 (9.7)	0	0.554
Liver disease, *n* (%)	1 (2.4)	1 (3.2)	0	1.000
Prior venous thromboembolism, *n* (%)	3 (7.1)	2 (6.5)	1 (9.1)	1.000
Autoimmune disease, *n* (%)	2 (4.8)	1 (3.2)	1 (9.1)	0.460
Hypothyroidism, *n* (%)	3 (7.1)	2 (6.5)	1 (9.1)	1.000
Chronic medications				
ACE-I/ARB, *n* (%)	10 (23.8)	2 (6.5)	8 (72.7)	<0.001
β-blockers, *n* (%)	11 (26.2)	5 (16.1)	6 (54.5)	0.021
Calcium channel blockers, *n* (%)	3 (7.1)	2 (6.5)	1 (9.1)	1.000
Anti-platelets, *n* (%)	10 (23.8)	4 (12.9)	6 (54.5)	0.011
Oral anticoagulation, *n* (%)	5 (11.9)	2 (6.5)	3 (27.3)	0.103
Diuretics, *n* (%)	2 (4.8)	1 (3.2)	1 (9.1)	0.460
Inhalations, *n* (%)	4 (9.5)	4 (12.9)	0	0.558
SGLT2 inhibitors, *n* (%)	1 (2.4)	0	1 (9.1)	0.262
Statins, *n* (%)	13 (31.0)	5 (16.1)	8 (72.7)	0.001
COVID-19 presentation				
Chest pain, *n* (%)	17 (40.5)	10 (32.3)	7 (63.6)	0.086
Shortness of breath, *n* (%)	25 (59.5)	15 (48.4)	10 (90.9)	0.016
Heart rate (bpm), mean ± SD	92.2 ± 21.1	91.8 ± 19.7	93.6 ± 35.0	0.632
SBP (mmHg), mean ± SD	123.0 ± 18.9	122.8 ± 18.6	123.3 ± 20.5	0.978
DBP (mmHg), mean ± SD	75.5 ± 12.7	75.1 ± 11.1	76.6 ± 17.1	0.800
Oxygen saturation (%), mean ± SD	90.3 ± 5.5	91.2 ± 5.1	87.7 ± 5.9	0.092
In-hospital course				
Sinus tachycardia, *n* (%)	5 (11.9)	3 (9.7)	2 (18.2)	0.593
Electrocardiogram changes				0.078
Normal, *n* (%)	25 (59.5)	20 (64.5)	5 (45.5)	
Nonspecific changes, *n* (%)	10 (23.8)	8 (25.8)	2 (18.2)	
TWI/ST-depression, *n* (%)	5 (11.9)	3 (9.7)	2 (18.2)	
ST-elevation, *n* (%)	2 (4.8)	0	2 (18.2)	
Chest X-ray infiltrates, *n* (%)	28 (66.7)	18 (58.1)	10 (90.9)	0.067
Atrial fibrillation/flutter, *n* (%)	4 (9.5)	0	4 (36.4)	0.003
Lab results				
White blood cells (peak), mean ± SD	8.6 ± 3.5	8.1 ± 2.8	10.0 ± 4.9	0.322
ANC/ALC (admission), median [IQR]	4.2 [2.6–8.6]	3.4 [2.4–6.9]	6.5 [4.8–9.7]	0.086
Hemoglobin (admission), mean ± SD	12.8 ± 2.3	13.2 ± 2.1	11.7 ± 2.5	0.110
Platelets (admission), mean ± SD	193.6 ± 55.7	192.7 ± 58.7	196.1 ± 49.0	0.591
Creatinine (admission), mean ± SD	1.0 ± 0.8	0.8 ± 0.3	1.6 ± 1.4	0.005
Potassium (admission), mean ± SD	4.0 ± 0.6	3.8 ± 0.4	4.4 ± 0.9	0.051
Albumin (trough), mean ± SD	3.4 ± 0.7	3.6 ± 0.6	2.8 ± 0.7	0.010
Hs-cTnI (peak), median [IQR]	6.5 [5.0–17.3]	5 [5–8.5]	40 [13.5–7467]	<0.001
CRP (peak), median [IQR]	6.5 [1.2–15.3]	3.6 [0.7–9.8]	21.4 [12.7–23.3]	<0.001
D-dimer (peak), median [IQR]	824 [549–1060]	728 [402–977]	1073 [921–1932]	0.001
Fibrinogen (admission), mean ± SD	578.7 ± 163.6	555.9 ± 151.8	640.9 ± 185.4	0.226
aPTT (peak), mean ± SD	3.3 ± 4.9	33.3 ± 5.1	33.3 ± 4.3	0.717
Exam characteristics and technical aspects				
Ability to turn left, *n* (%)	35 (83.3)	28 (90.3)	7 (63.6)	0.063
Effective communication, *n* (%)	39 (92.9)	30 (96.8)	9 (81.8)	0.163
Length of study (minutes), mean ± SD	6.8 ± 2.2	6.4 ± 2.1	8.1 ± 2.0	0.007
Full view successful completion, *n* (%)	38 (90.5)	29 (93.5)	9 (81.8)	0.277
Study quality				0.618
Good, *n* (%)	27 (64.3)	19 (61.3)	8 (72.7)	
Fair, *n* (%)	8 (19.0)	7 (22.6)	1 (9.1)	
Poor, *n* (%)	7 (16.7)	5 (16.1)	2 (18.2)	

Abbreviations. ACE-I, angiotensin-converting enzyme; AI, artificial intelligence; ALC, absolute lymphocyte count; ANC, absolute neutrophile count; aPTT, activated partial thromboplastin time; ARB, angiotensin II receptor blocker; bpm, beats per minute; CIED, cardiovascular implantable electronic device; cm, centimeters; CRP, C-reactive protein; DBP, diastolic blood pressure; Hs-cTnI, high-sensitivity cardiac troponin I; IQR, interquartile range; mmHg, millimeter of mercury; LVEF, left ventricular ejection fraction; *n*, number; SD, standard deviation; SBP, systolic blood pressure; SGLT2, sodium-glucose transport protein 2; TWI, T-wave inversion.

### 3.2. LVEF Assessment Correlations and Agreement

**Figure 2:**
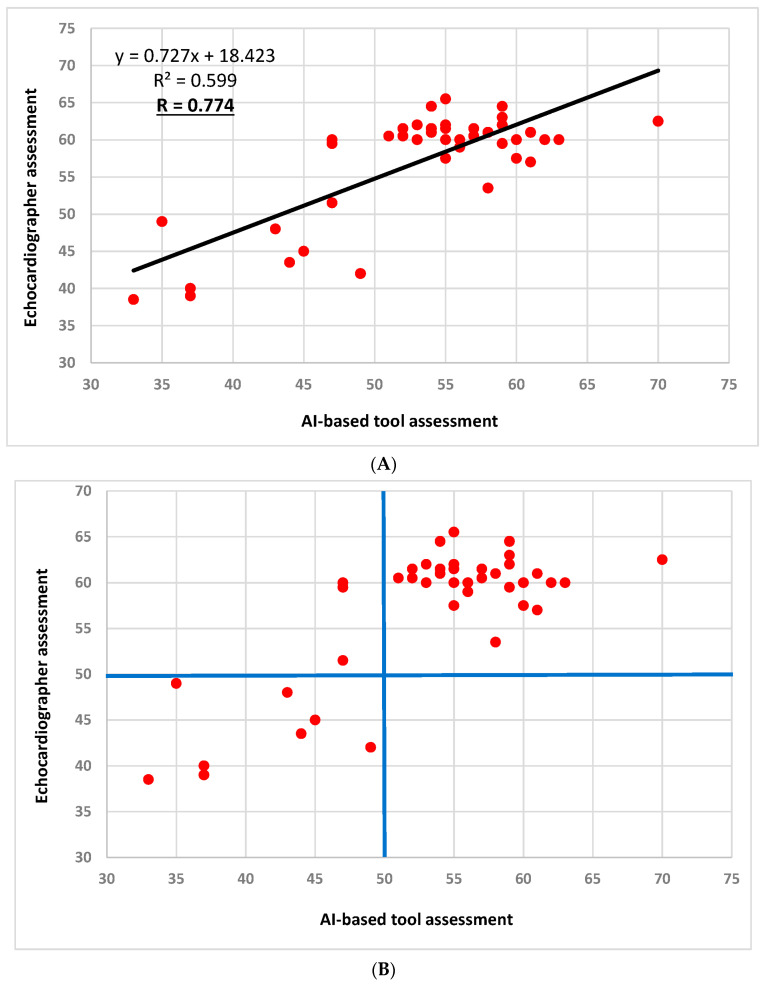
LVEF assessment correlation and agreement between the AI-based tool and the echocardiographer (two patients had identical assessments that are superimposed: 60 vs. 55, respectively). (**A**): LVEF assessment correlation between the AI-based tool and the echocardiographer; Pearson’s correlation coefficient = 0.774 (*p* < 0.001). (**B**): LVEF assessment agreement between the AI-based tool and the echocardiographer using the LVEF cutoff of 50% (marked by the blue horizontal and longitudinal lines); Kappa coefficient = 0.797 (standard error of 0.110, *p* < 0.001). Abbreviations. AI, artificial intelligence; LVEF, left ventricular ejection fraction.

Excellent correlation was demonstrated between the AI and the echocardiographer LVEF assessment (Pearson’s correlation of 0.774, *p* < 0.001, Figure 2A). Substantial agreement was demonstrated between the AI and the echocardiographer for LVEF using a threshold of 50% (kappa = 0.797, *p* < 0.001, Figure 2B). 

### 3.3. Sensitivity and Specificity Analyses

The sensitivity, specificity, PPV, and NPV of the HUD using the AI-based tool for the 50% LVEF threshold were 72.7%, 100%, 100%, and 91.2%, respectively.

### 3.4. Association between Reduced LVEF (<50%) Using the AI-Based Tool and Study Endpoints (Table 2 and Figure 3)

The AI-based diagnosis of reduced vs. preserved LVEF was associated with the endpoints of myocardial injury, ADHF, and acute kidney injury, and with the composite outcome, including in-hospital death, advanced ventilatory support, shock, myocardial injury, and ADHF (72.7% vs. 19.4%, respectively, *p* = 0.003, unadjusted OR = 11.11 with 95% CI 2.25–54.94). Multivariate analysis adjusting for age and combined pertinent comorbidities revealed that AI-based reduced LVEF was independently associated with the composite endpoint OR = 6.40 (95% CI 1.07–38.09, *p* = 0.041).

**Figure 3:**
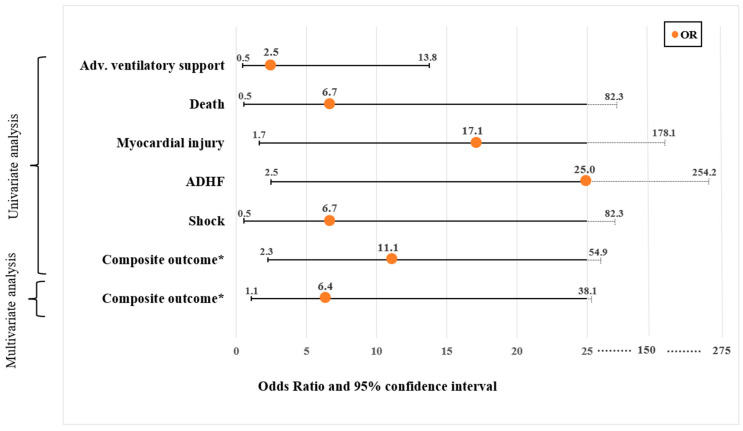
Significant associations (odds ratio and 95% confidence interval^+^) between AI-based reduced left ventricular systolic function (LVEF < 50%) and serious adverse events (endpoints). The figure includes the univariate associations as well as multivariate logistic regression analysis results that were calculated to test the correlation between reduced LVEF and the composite and individual endpoints. The multivariate logistic regression analysis included age and combined pertinent comorbidities variables (baseline characteristic covariates with *p* values of less than 0.05). The odds ratios (ORs) are illustrated by the orange dots, while the line represents the 95% confidence interval (CI). The values in the *x*-axis are presented in a linear fashion up to 25. From this point on, the progression deviates from linearity. + Numeric results of OR and 95% CI and are detailed in Table 2. * The primary endpoint was defined as a composite endpoint of in-hospital death, advanced ventilatory support, shock, myocardial injury, and acute decompensated heart failure. Abbreviations. ADHF, acute decompensated heart failure; Adv., advanced; AI, artificial intelligence; LVEF, left ventricular ejection fraction.

**Table 2:** The association between reduced LVEF as per the AI-based tool and serious adverse events (endpoints).

Variable	All *n* = 42	Preserved LVEF *n* = 31	Reduced LVEF *n* = 11	*p*-Value	Unadjusted OR (95% CI)
Composite endpoint, *n* (%)	14 (33.3)	6 (19.4)	8 (72.7)	0.003	11.1 (2.25–54.94)
In-hospital death, *n* (%)	2 (4.8)	0	2 (18.2)	0.064	6.7 (0.54–82.31)
Advanced VS, *n* (%)	7 (16.7)	4 (12.9)	3 (27.3)	0.282	2.5 (0.47–13.75)
Myocardial injury, *n* (%)	5 (11.9)	1 (3.2)	4 (36.4)	0.017	17.1 (1.65–178.08)
Shock, *n* (%)	2 (4.8)	0	2 (18.2)	0.064	6.7 (0.54–82.31)
ADHF, *n* (%)	6 (14.3)	1 (3.2)	5 (45.5)	0.007	25 (2.46–254.15)
RRT, *n* (%)	2 (4.8)	0	2 (18.2)	0.064	6.7 (0.54–82.31)
VTE, *n* (%)	1 (2.4)	1 (3.2)	0	1.000	3.0 (0.17–52.53)
Anti-COVID drugs, *n* (%)	18 (42.9)	12 (38.7)	6 (54.5)	0.362	1.9 (0.47–7.63)
Sepsis, *n* (%)	2 (4.8)	0	2 (18.2)	0.064	6.7 (0.54–82.31)
Acute kidney injury, *n* (%)	6 (14.3)	5 (16.1)	1 (9.1)	0.007	25.0 (2.46–254.15)
LOS (days), median [IQR]	5.1 [2.7–10.4]	4.8 [2.2–10.1]	6 [4.3–12.4]	0.201	
LOS > median (5.1 days), *n* (%)	21 (50.0)	16 (51.6)	5 (45.5)	0.726	1.28 (0.32–5.01)

Abbreviations. ADHF, acute decompensated heart failure; CI, confidence interval; IQR, interquartile range; LOS, length of stay; LVEF, left ventricular ejection fraction; *n*, number; OR, odds ratio; RRT, renal replacement therapy; VS, ventilatory support; VTE, venous thromboembolism.

## 4. Discussion

The current prospective study shows that among non-selected hospitalized COVID-19 patients, AI-based tool use on an HUD operated by clinicians is highly correlated to expert echocardiographers and provides a real-time accurate LVEF measurement over a range of cardiac functions. Moreover, the AI measurement can reach a high level of agreement with echocardiographers for the diagnosis of reduced LV systolic function (LVEF < 50%) with 100% sensitivity and PPV, very high NPV, and satisfactory specificity. Patients admitted with COVID-19 with reduced LVEF based on the AI tool assessment were older and presented with a higher burden of comorbidities, worse laboratory results, and a more prolonged exam and the closer proximity of the examiner to the patients. The study also reveals an association between AI-based reduced LV systolic function and worse composite endpoints.

HUD use has gained popularity and expanded across medical disciplines due to their many advantages, including small sizes, portability, low cost, and instantaneous assessment. These characteristics are useful in the COVID-19 setting, with a possible direct impact on immediate patient diagnosis and management [12]. Given the objective difficulties associated with the COVID-19 working environment for clinicians [16], real-time LVEF assessment can be challenging. In this context, this AI tool can provide the operator with rapid LVEF quantification, including the immediate visualization of its endocardial border tracking, as well as a tool for the risk stratification of these patients. Multiple software programs have incorporated automation to improve the accuracy and efficiency of manual tracings alongside different automated measurements [17]. A different automated tool for the evaluation of LV function has been validated by Asch et al., using echocardiographers, assuming the left ventricle contracts throughout the cardiac cycle along its long and short axis to produce an accurate LVEF from proportional changes in cardiac chamber size [18]. Unlike the current AI-based tool, their tool estimates LVEF without quantifying the end-systolic and diastolic volumes.

The accuracy of AI-based LVEF assessment has been recently evaluated by comparing AI-generated LVEF measurements vs. a sonographer’s tracings as compared to a final LVEF assessment by a cardiologist [19]. It was shown that the AI algorithm was non-inferior and even superior to the sonographer’s assessment. Also, and similar to the current study’s findings, Samtani et al. have validated this AI-based tool, LVivo EF, using clips acquired by a traditional echocardiogram device in a prospective study that included 242 patients, demonstrating that it can accurately quantify LVEF as compared with cardiac MRI without manual correction with a Pearson’s correlation of 0.89 [20]. Also, Filipiak-Strzecka et al. tested this AI tool on HUD-acquired clips (measurement was successful in 76 patients out of 112) by a trained cardiology resident, revealing an excellent correlation of 0.92 as compared with a high-end ultrasound device operated by an accredited echocardiographer [15]. The slightly higher correlation presented in these two aforementioned studies as compared with the present study may stem from the COVID-19 environment leading to a less convenient setting, resulting in lower exam quality.

With regard to measurement success, Samtani et al. have shown that this AI tool was unsuccessful in measuring the LVEF in 2.5% of the attempted tests (6/242 patients). Conversely, the rate of unsuccessful measurement in the present study was higher (~19%; 10/52 patients). This discrepancy may stem from the different ultrasound devices used for the echocardiogram clip acquisition and the study setting; whereas Samtani et al. used a high-end device on cardiac patients among non-COVID 19 patients, the present study used HUDs on patients admitted with COVID-19. Indeed, Filipiak-Strzecka et al. showed an unsuccessful measurement rate that was higher (32.1%; 36/112 exams) using an HUD, with a smaller number of attempts for each exam (three attempts).

The current study also shows that reduced LV systolic function based on AI assessment is associated with worse endpoints and is predictive of the composite endpoints of in-hospital death, advanced ventilatory support, shock, myocardial injury, and ADHF. To the best of our knowledge, no previous studies have investigated the association of AI automated echocardiogram indices with patient outcomes. An association between abnormal echocardiogram results, including valvular pathologies and reduced LVEF, and outcomes has been previously demonstrated by our group in a recent prospective study, revealing an independent association with the study composite endpoints, including death, mechanical ventilation, shock, and ADHF, supporting the current study’s findings [12].

### Limitations

This prospective research was conducted at a single site and may be subjected to the relevant confounders. The populations and the quality of the exams may differ between hospitals. This may limit the generalizability of the findings. Also, while it is correlated with the study design and sample size calculation, the limited size of the cohort may expose it to biases. Furthermore, similar to other studies but with differing rates, the present research also reported a rate of unsuccessful measurements of 19% by the AI-based tool and also included up to four assessment attempts [15,20]. This limited generalizability is the “cost” of conducting a study in clinical settings, especially when it comes to a pandemic and the extreme precautions taken, including wearing a gown, mask, and gloves. Nonetheless, for patients in whom the AI-based tool was successful, we found reassuring accuracy as well as adjusted correlation with unfavorable outcomes.

## 5. Conclusions

This AI-based algorithm incorporated into an existing HUD can be reliably utilized as a decision support tool with high diagnostic performance for automatic real-time LVEF assessment among hospitalized COVID-19 patients. LVEF dysfunction based on this AI tool can identify patients with detrimental baseline and medical characteristics with more challenging echocardiogram exam features that may be at higher risk for unfavorable outcomes, thus providing a complementary tool to physicians to enhance patient care. Larger cohorts should be studied to examine whether AI-based reduced LVEF is associated with unfavorable outcomes. 

## Data Availability

Data may be available for sharing.
